# Impact of Pre- and Post-therapeutic Exercises in Sarcopenia and Pain in Liver Transplant Patients

**DOI:** 10.7759/cureus.64360

**Published:** 2024-07-11

**Authors:** Abdullah N AlShahrani, Thamir M Al-Khlaiwi, Sultan A Meo

**Affiliations:** 1 Department of Physiology, College of Medicine, King Saud University, Riyadh, SAU

**Keywords:** pain, psoas major muscle, exercises, liver transplantation, sarcopenia

## Abstract

Introduction: Sarcopenia, a state considered by the loss of muscle function and mass, is progressively recognized as a common complication of advanced cirrhosis and is related to negative clinical consequences. Liver transplantation (LT) is one of the options in the treatment of cirrhosis. This study aimed to evaluate the impact of exercise in newly developed (ND) sarcopenia through measurement of the psoas major muscle at the level of the third lumbar vertebra by abdominal computed tomography (CT) images in liver transplant patients.

Method: This analytical case-control longitudinal study was conducted on patients aged between 16 and 70 years at King Fahad Specialist Hospital (KFSH-D) in Dammam, Saudi Arabia. The patients were divided into two groups: an intervention group consisting of liver transplant patients (LTx, n=26) and a control group consisting of end-stage liver disease patients (ESLD, n=23) who were not candidates for LT. All participants were treated with a therapeutic exercise program through three phases. The first phase included pre-operative exercises, the second phase included early post-operative exercises, and the last phase included late post-operative exercises. CT scan of the psoas major muscle was performed pre- and post-LT to assess sarcopenia. Pain was measured with a numerical pain scale before and after exercise to evaluate the impact of exercise on pain.

Result: Psoas muscle index (PMI) for post-therapeutic exercises in the control and liver transplant groups showed significant differences for both male and female patients compared with the normal range (p<0.05). The liver transplant group showed a significant difference in mean weight loss, body mass index (BMI), and numerical pain rating scale (NPRS) observed on post-therapeutic exercises compared to baseline data.

Conclusion: By and large, the finding revealed a substantial impact of therapeutic exercise on patient outcomes in terms of anthropometric characteristics, abdominal pain, handgrip strength (HGS), and PMI. The mean PMI at post-therapeutic exercises showed a significant increase by measuring the psoas major muscle at the level of the third lumbar vertebra by abdominal CT, which may indicate the extent of improvement and recovery from the ND sarcopenia in LTx. The results emphasize the potential advantages of pre- and post-therapeutic exercise in LTx, including enhancements in muscle strength and mass, as well as pain management associated with liver function and general health. Optimizing patient outcomes and promoting a more comprehensive approach to liver disease treatment may be achieved by including exercise activities in pre- and post-LT care procedures.

## Introduction

Sarcopenia, a state considered by loss of muscle function and mass, is progressively recognized as a common complication of advanced cirrhosis and is related to negative clinical consequences [1,2]. Sarcopenia and functional impairment are potentially life-threatening and could extend their impact on various aspects of life [3]. The sarcopenia prevalence in end-stage liver disease (ESLD) patients varies from 10% to 70%, depending on the severity of hepatic dysfunction [4]. Patients with sarcopenia show reduced muscle strength and low skeletal muscle mass, which results in muscle atrophy and physical disabilities, which is a major concern [4]. Computed tomography (CT) is a method that provides an objective anatomical assessment of muscle changes and consequently sarcopenia. However, using whole-body CT for repeated and routine body composition assessment is impossible. In its place, measuring the thickness of the psoas muscle on a CT scan has been used as an approximation of muscle mass and hence sarcopenia [2]. This technique involves determining the cross-sectional area of muscle at the level of the third (L3) or fourth (L4) lumbar vertebrae, which has shown a good association with total body muscle mass [1]. Nishikawa studied the usefulness of the psoas muscle index (PMI) to evaluate muscle mass in healthy donors for living donor liver transplants. The PMI was achieved by evaluating the iliopsoas muscle area at the level of L3 through a CT scan and dividing it by the height squared [5].

Handgrip strength (HGS), as a functional assessment of sarcopenia, was found to be a simple method and noninvasive approach for evaluating the strength of muscle. The research data confirmed that using an HGS as a standalone tool for showing low muscle mass was highly effective [4]. According to the criteria of Japan Society of Hepatology (JSH) guidelines for sarcopenia in liver disease, values of HGS below 18 kg in females and 26 kg in males were considered low HGS showing limited muscle strength [4].

Executing exercise programs before surgery can enhance both quality of life and muscle strength for hospitalized patients awaiting liver transplantation (LT). Furthermore, therapeutic exercises before transplantation can help patients reduce post-operative complications [6,7]. Consequently, the incorporation of well-designed physiotherapy initiatives holds promise in mitigating functional losses and optimizing outcomes in the LT [8]. After the liver transplant surgery, therapeutic exercises are started as part of the patient's early care of post-transplant. These exercises emphasize mobilization and physiotherapy from the first day after the operation until the patient is discharged from the intensive care unit (ICU) [7]. Roshdy et al. have conducted research aimed at evaluating the effect of early pulmonary rehabilitation following a liver transplant [9]. Immediate post-operative care is necessary following liver transplant surgery and should continue for three months after the patient is discharged to preserve optimal physical activity [7] and is essential in long-term health outcomes [10]. Finally, in a comprehensive overview of ongoing care and management of pre- and post-LT, Dhaliwal and Raghunathan observed that early LT has risen as the definitive and established treatment for ESLD in the last few decades and post-operative intensive care has significantly contributed to enhanced survival rates and overall outcomes [11].

This study aimed to assess the impact of exercise in sarcopenia through measurement of the psoas major muscle at the level of the third lumbar vertebra by abdominal CT images and pain in patients with liver transplants.

## Materials and methods

This study is an analytical case-control longitudinal study which was conducted at King Fahad Specialist Hospital (KFSH-D) in Dammam, Saudi Arabia. The study period lasted for two years from the beginning of the Ethics Committee (IRB) approval on November 1, 2021. A total of 49 adult patients by using convenience sampling were distributed in two groups, with 26 undergoing LT and 23 serving as a control group. All the participants including intervention and control groups were between the ages of 18 and 70 years in both genders. These groups were separated according to their liver disease stage and whether the patient has either LTx or ESLD within KFSH in Dammam. In contrast, individuals with recent myocardial infarction, uncontrolled arrhythmias, acute pulmonary embolism, uncontrolled bleeding disorders, or active infections, or with advanced cardiovascular or respiratory disease or other severe medical conditions that could pose a risk during exercise, and with significant cognitive impairments or physical limitations that hinder their ability to understand and follow the exercise program or engage in physical activities were excluded from the study.

Pre- and post-operative exercises

Participants received a therapeutic exercise program by the design of the Swiss Program of Physical Activity pre- and post-LT in phases [12], starting with the first phase and continuing until the end of the third phase.

Phase 1: Pre-operative Exercises

Therapeutic exercises were administered for one month before surgery. Exercises were used as part of the pre-transplant care at our center with pre-operative education for both groups that received the following standard supervised physiotherapy follow-up [13].

The following were the exercises implemented three times per week: (1) stretching exercises to increase flexibility and range of motion in the muscles and joints, (2) strengthening exercises to increase muscle strength, which can lead to better post-operative outcomes and faster recovery, and (3) cardiovascular exercise (walking). It is essential in pre-operative preparation to improve cardiovascular fitness and overall health. Participants received instruction on how to deal with walking a home-based exercise program (HBEP), considering their individual rehabilitation requirements and the principles of graded activity. Weekly follow-up calls were made to keep up the walking and schedule follow-up appointments.

Phase 2: Early Post-operative Exercises

The following were the therapeutic exercises employed in the ICU and then in the transplant unit daily for two weeks [7,12]: First is pulmonary physiotherapy: we can use forced expiratory and coughing techniques (16) or an instrument called an incentive spirometer device, where the patient breathes as deeply as he can through a tube connected to a hand-held plastic chamber. This chamber contains three balls that rise with each breath. This method is performed 5-10 consecutive times every hour while the patient is awake. Second is gradually introducing mobilization stretching exercises for the patient's limbs: they start with limb exercises while the patient is supine and progress to sitting in bed, standing, and finally walking exercises. It is important to keep in mind that all exercises are closely supervised.

Phase 3: Late Post-operative Exercises

The following therapeutic exercises were applied in the first three months after surgery and implemented three times per week [7,12]: (1) stretching exercises and (2) strengthening exercises.

Data collection

Data were collected before applying pre-operative exercises one month before LT and three months after LT after applying post-operative exercises for the experimental group. For the control group, data was collected before applying therapeutic exercises and after four months after the completed therapeutic exercises.

Clinical examinations were performed on all patients with chronic liver diseases deemed candidates for LT including body weight (BW) and height (Ht) to calculate body mass index (BMI), pain assessment by numerical pain rating scale (NPRS), and handgrip test to assess sarcopenia.

**Figure 1:**
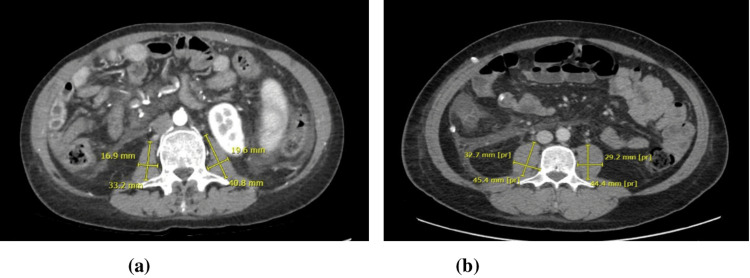
Psoas major muscle assessment images in a liver transplant patient. (a) Abdominal CT image (horizontal section) showing the third lumbar vertebra to calculate the PMI for pre-therapeutic exercises. (b) Abdominal CT image (horizontal section) showing the third lumbar vertebra to calculate the PMI for post-therapeutic exercises. CT: computed tomography; PMI: psoas muscle index

Radiological examinations were done using abdominal CT images at the level of the third lumbar vertebra to assess sarcopenia through measurement of the psoas major muscle. The XERO® software application was utilized to measure and identify the psoas major muscle using imaging data (Figure 1). This software was used to calculate the PMI value using a simple method. We utilized the PMI to assess sarcopenia.

Data analysis

The categorical response variable was presented as frequency and percentage. A chi-squared test was applied to compare control and liver transplant groups. All the continuous variables like age, weight, height, BMI, NPRS, HGS, and PMI were presented as mean±standard deviation. Kolmogorov-Simonov's test of normality was applied to test the distribution of data. Most of the variables were normally distributed. A paired samples t-test was applied to compare the mean of baseline demographic characteristics and psoas major muscle measurements at the L3 level as pre-therapeutic exercises (Exs) versus post-Exs within control and liver transplant groups. The effect of post-Exs between the control and liver transplant group was compared by using an unpaired t-test. A p-value less than or equal to 0.05 was considered statistically significant. In both the control and the liver transplant groups, the post-Exs effect relationship between HGS and PMI was shown by calculating the Pearson correlation coefficient (r) and making scatter plots.

## Results

A total of 49 patients participated in the study. Out of them, 26 (53.1%) underwent LT, whereas 23 (46.9%) patients were considered as controls who were observed on the waiting list for LT as per the study protocol. While comparing demographic data including the mean age, weight, height, BMI, NPRS, and HGS between control and liver transplant groups, non-significant differences were seen for baseline data at a 5% level of significance. However, significant mean differences in BMI and NPRS were seen for post-therapeutic exercise data between control and liver transplant groups (Table 1).

**Table 1:** Comparison of demographic characteristics of the baseline and post-therapeutic exercises and between control and liver transplant groups. *A significant difference in mean at a 5% level of significance. ^a^Sig: a significant value for the mean difference between pre-Exs and post-Exs within the control group and the liver transplant group. ^b^Sig: a significant value for the mean difference between the control group and the liver transplant group. Exs: therapeutic exercises; HGS: handgrip strength; NPRS: numeric pain rating score (abdominal pain)

Demographic characteristics	Control (n=23)		^a^Sig.	Liver transplant (n=26)		^a^Sig.	^b^Sig.	^b^Sig.
	Pre-Exs	Post-Exs		Pre-Exs	Post-Exs		Pre-Exs	Post-Exs
Age (years)	49.4±10.9	-	-	52.0±12.7	-	-	0.449	-
Weight (kg)	77.3±16.7	75.7±16.4	0.128	74.1±14.0	68.4±12.7*	0.001	0.471	0.086
Height (m)	1.62±0.11	1.61±0.10	0.069	1.65±0.09	1.65±0.09	0.664	0.265	0.156
BMI (kg/m^2^)	29.5±5.9	29.1±6.0	0.372	27.1±4.5	25.0±4.5*	0.001	0.112	0.009
NPRS	3.5±2.1	4.4±2.6*	0.032	3.8±1.3	0.92±1.0*	0.001	0.619	0.001
HGS (kg)	21.4±8.3	23.0±6.9	0.091	24.1±12.1	22.7±9.7	0.485	0.375	0.888

The control group did not expose any significant differences of therapeutic exercises on weight, BMI, and HS except that the mean NPRS significantly increased (p=0.032) on post-therapeutic exercises (4.4±2.6) compared to pre-therapeutic exercises (3.5±2.1), whereas, in the liver transplant group, a significant difference in mean weight loss, BMI, and NPRS was observed on post-therapeutic exercises compared to baseline data (Table 1).

Based on the JSH guidelines, we compared the findings to the normal range of the HGS and PMI. The mean HGS in pre- and post-therapeutic exercises in the control group revealed non-significant differences for male patients p=0.577 and p=0.878, respectively. The reference range of HGS for female patients was ≥18 kg compared to the mean HGS in pre- and post-therapeutic exercises followed by the likewise non-significant differences for female patients as detailed in Table 2. There was a statistically significant difference (p=0.040) between the mean PMI in post-therapeutic exercises and the normal range of PMI for male patients in the control group, but there was no significant difference between the mean PMI in pre-therapeutic exercises and the normal range of PMI for male patients, which was 6.36 cm^2^/m^2^. Furthermore, when comparing the pre-therapeutic exercises with the normal range of PMI for female patients, which is ≥3.93 cm^2^/m^2^, the mean PMI at baseline was significantly greater (p=0.006), but it was not significant after the post-therapeutic exercises in the control group (Table 2).

**Table 2:** Comparison of gender-specific mean HGS and PMI pre- and post-therapeutic exercises based on normal reference value in the control group. *A significant difference in the mean based on normal reference value at a 5% level of significance. ^a^A significance value for the difference of pre-Exs with normal range. ^b^A significance value for the difference of post-Exs with normal range. Exs: therapeutic exercises; HGS: handgrip strength; PMI: psoas muscle index PMI at L3=(total psoas muscle area of L3 level (cm^2^))/(height(m)×height(m))

Variable	Male				Female			
	Normal range	Pre-Exs	Post-Exs	Sig.	Normal range	Pre-Exs	Post-Exs	Sig.
HGS (kg)	≥26.0	24.7±8.4	25.7±6.8	^a^0.577; ^b^0.878	≥18	16.2±5.1	18.8±4.7	^a^0.323; ^b^0.636
PMI (cm^2^/m^2^)	≥6.36	7.6±2.6	7.8±2.2*	^a^0.105; ^b^0.040	≥3.93	6.2±1.7*	5.8±2.8	^a^0.006; ^b^0.132

In the liver transplant group, the mean HGS in pre- and post-therapeutic exercises revealed non-significant differences for male patients with a significant decrease in mean HGS for female patients, as detailed in Table 3. In addition, the mean PMI in pre- and post-therapeutic exercises were compared with the normal range that have shown significantly higher mean PMI in both male and female patients (p<0.05) as detailed in Table 3.

**Table 3:** Comparison of gender-specific mean HGS and PMI pre- and post-therapeutic exercises based on normal reference value in the liver transplant group. *A significant difference in the mean based on normal reference value at a 5% level of significance. ^a^A significance value for the difference of pre-Exs with normal range. ^b^A significance value for the difference of post-Exs with normal range. Exs: therapeutic exercises; HGS: handgrip strength; PMI: psoas muscle index PMI at L3=(total psoas muscle area of L3 level (cm^2^))/(height(m)×height(m))

Variable	Male				Female			
	Normal range	Pre-Exs	Post-Exs	Sig.	Normal range	Pre-Exs	Post-Exs	Sig.
HGS (kg)	≥26.0	29.4±11.5	27.2±8.6	^a^0.238; ^b^0.562	≥18	14.0±3.9*	14.0±4.3*	^a^0.016; ^b^0.024
PMI (cm^2^/m^2^)	≥6.36	8.5±3.1*	8.9±2.7*	^a^0.014; ^b^0.016	≥3.93	5.0±1.6*	7.1±2.1*	^a^0.033; ^b^0.007

**Figure 2:**
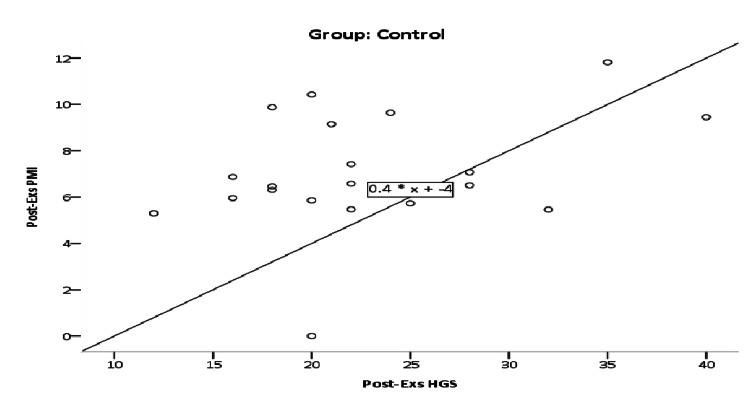
Scatter plot of post-therapeutic exercises relationship between HGS and PMI in the control group. HGS: handgrip strength; PMI: psoas muscle index

**Figure 3:**
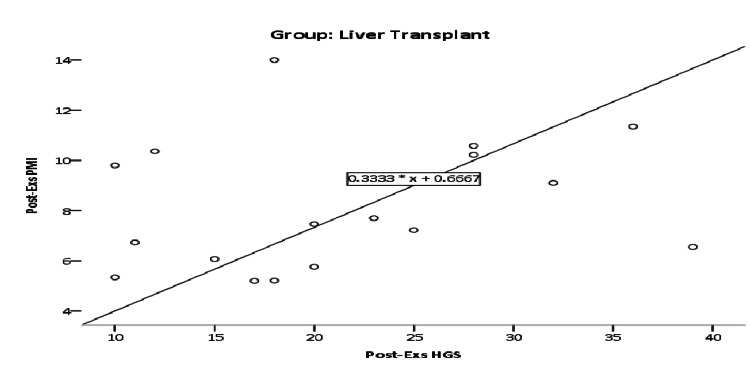
Scatter plot of post-therapeutic exercises relationship between HGS and PMI in the liver transplant group. HGS: handgrip strength; PMI: psoas muscle index

Post-therapeutic exercise's effect relationship between HGS and PMI in the control group was r=0.343; however, this positive correlation was non-significant (p=0.139), as illustrated in Figure 2. Post-therapeutic exercise's effect relationship between HGS and PMI in the liver transplant group was r=0.225; however, this positive correlation was non-significant (p=0.386) as illustrated in Figure 3.

**Figure 4:**
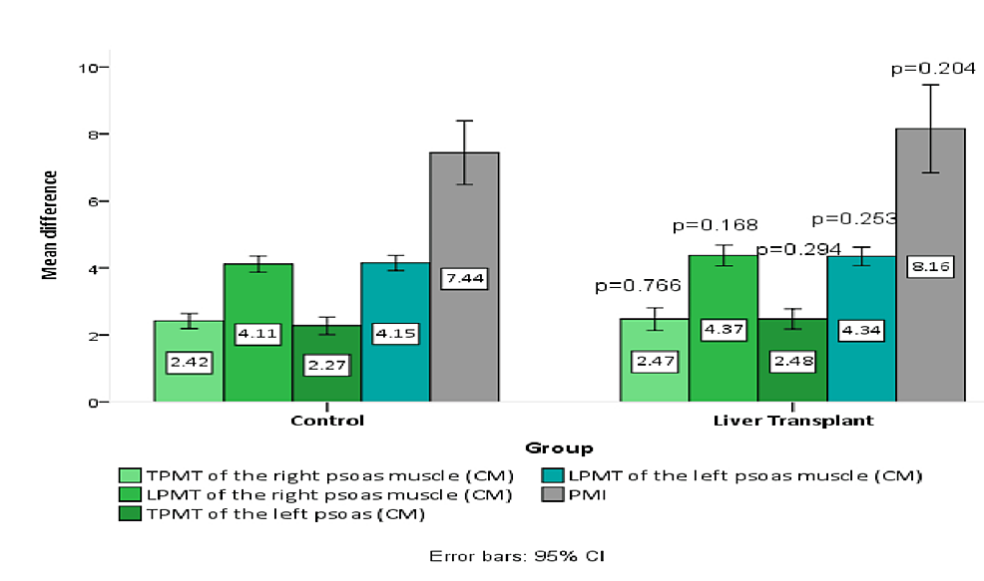
Comparison of post-therapeutic exercise effect on mean psoas major muscle measurements at L3 level between the control and liver transplant group.

Only the mean transverse psoas muscle thickness (TPMT) of the right psoas muscle was found significant after therapeutic exercise in the control group (p=0.049), while the non-significant effect of post-therapeutic exercise was seen with regard to mean psoas major muscle measurements at L3 level including PMI in the control and liver transplant groups (Table 4). Post-therapeutic exercise effect between the control and liver transplant group was also non-significant (p>0.05) as presented in Figure 4.

**Table 4:** Comparison of pre- versus post-therapeutic exercises effect on mean psoas major muscle measurements at L3 level between the control and liver transplant group. *A significant difference in mean between the control and liver transplant group at a 5% level of significance. Exs: therapeutic exercises; TMMT: transverse psoas muscle thickness; LPMT: longitudinal psoas muscle thickness PMI at L3=(total psoas muscle area of L3 level (cm^2^))/(height(m)×height(m))

Psoas major muscle measurements at the L3 level	Control (n=23)			Liver transplant (n=26)		
	Pre-Exs	Post-Exs	Sig.	Pre-Exs	Post-Exs	Sig.
TPMT of the right psoas muscle (CM)	2.21±0.58	2.42±0.47*	0.049	2.20±0.69	2.47±0.65	0.088
LPMT of the right psoas muscle (CM)	4.10±0.54	4.11±0.50	0.877	4.22±0.67	4.37±0.62	0.154
TPMT of the left psoas muscle (CM)	2.20±0.58	2.27±0.54	0.557	2.27±0.65	2.48±0.59	0.308
LPMT of the left psoas muscle (CM)	4.17±0.74	4.15±0.48	0.863	4.30±0.71	4.34±0.54	0.368
PMI (cm^2^/m^2^)	7.04±2.32	7.07±2.54	0.939	7.13±2.71	8.16±2.56	0.148

## Discussion

This study aimed to measure sarcopenia through the anatomical measurement of the psoas major muscle at the level of the third lumbar vertebra by abdominal CT images as well as HGS pre- and post-operative exercises in LTx identifying the potential benefits of exercise in liver disease management. Patients undergoing LT face significant physiological changes, and therapeutic exercises may offer a proactive method for post-transplant recovery and overall well-being.

Post-therapeutic exercises exposed significant changes in BMI, weight loss, and pain scores in the liver transplant group compared to baseline, whereas the control group showed significant changes only in pain scores post-therapeutic exercises.

HGS and PMI measurements exhibited varied responses to exercises between the groups, with notable differences in PMI for both male and female patients' post-therapeutic exercises in the control and liver transplant groups. The observed changes in BMI, weight, HGS, pain scores, and PMI markers post-therapeutic exercises recommend that exercise interventions may have a valuable impact on liver transplant patients. Whereas both groups experienced some improvements, the liver transplant group showed more significant changes, potentially indicating a greater need for responsiveness to exercise interventions in this population. The correlation between PMI and HGS mentioned the interplay between mass and muscle strength, recommending that improvements in one may positively impact the other.

Despite the deliberate or appropriate selection of patients for LT following the donor's availability, the recipient's HLA matching, and their overall medical fitness for the procedure, the two groups were matched in terms of gender. Though the liver transplant group is older, with a mean age of 52+/-12.7 years, the difference from the control group is not significant. The loss of weight measured at three months post-therapeutic exercises in the LTx group could be an indication of either sarcopenia, loss of water from ascites, or edema compared to the ESLD patients.

Surely, the control group was not as bedridden as the LTx group that was operated upon. Sarcopenia develops rapidly when older individuals are bedridden, even for short periods. This sarcopenia is even more rapid in older patients compared to younger ones, and the age range of the LTx was toward the older side (52.0±12.7) compared to the control group (49.4±10.9).

Though the handgrip test is not significant between pre- and post-therapeutic exercises in the LTx group, the hand grip is reduced at three months post-therapeutic exercises. This also goes with weight loss, which could be because of muscle loss (sarcopenia). The term "newly developed" (ND) sarcopenia refers to sarcopenia that develops one or two years after LTx in individuals who have never had sarcopenia before LT, according to a recent study published in 2023 [14]. To evaluate the degree of sarcopenia by using CT scans, this study compared pre-LTx and one- and two-year post-LTx periods. The average skeletal muscle area divided by height squared was used to determine sarcopenia. In comparison to patients with sarcopenia before LTx, people with ND sarcopenia had significantly worse results. The following conditions are potential causes of ND sarcopenia in individuals who have undergone LT: post-transplant infection, renal failure, immunosuppressive drugs, including corticosteroids and calcineurin inhibitors, and prolonged hypermetabolic hypertrophy [14].

PMI at L3 level increased at post-therapeutic exercises in both groups, even though there were non-significant differences compared to pre-therapeutic exercises. In addition, the mean PMI at post-therapeutic exercises was compared with the normal range, which showed significantly higher mean PMI in both groups (male and female). The result of PMI is a good sign that represents a preventive effect against muscle loss (sarcopenia) that is specifically important for older patients immobilized post-operation. These results highlight the potential rapid onset and extent of muscle loss within a short time limit in this demographic.

Aarden et al. found that being bedridden may lead to a loss of muscle mass and muscle strength. Muscle loss could appear very rapidly within a week to two weeks, and depending on the age of the patient, it could be quite extensive. As a result, it might affect muscle mass and muscle strength even more in older adults [15]. Furthermore, systemic infections are quite common following LT, since patients should be on immunosuppressants for the rest of their lives. These infections are linked to higher rates of mortality and morbidity, which can delay improvements in the muscle and functional capacity of the patients [16].

Usually, ESLD patients have ascites and general edema because the liver is not producing enough albumin protein to keep the water in circulation [17]. The two primary pathophysiological processes that cause ascites in people with cirrhosis are portal hypertension and salt and water retention [18].

Multiple pathophysiological pathways have been identified in the etiology of sarcopenia in individuals with liver cirrhosis, according to a recent review study published in 2023. There is a recurrent imbalance between the building and breaking down of muscles in liver cirrhosis. Sarcopenia in liver cirrhosis patients is categorized as secondary and has been linked to higher rates of mortality and morbidity both before and after transplantation [19].

The results highlight the potential benefits of pre- and post-therapeutic exercises in LTx, comprising improvements in muscle strength, mass, and pain management related to liver function and overall health. Integrating exercise interventions into pre- and post-LT care protocols may improve patient outcomes and contribute to a more holistic approach to liver disease management. In addition, expectations concerning total levels of protein intake and nutritional therapy may improve outcomes post-LT in nutrition and liver synthetic function [20]. A similar study found that patients awaiting LTx had a significant level of anxiety and depression (61%), which did not improve with home-based exercise for patients (HBEP) alone [21].

This study is subject to some limitations. It was not able to draw a broad conclusion from the study because of its very small sample size: just 26 patients received LT, and 23 patients completed the control group. The study's findings were impacted by confounding variables, including variations in age, comorbidities, use of medications, and transplant standards.

It would be extremely beneficial to do a large-scale study to determine the effects of exercise on sarcopenia by measuring the psoas major muscle by abdominal CT imaging at the level of the third lumbar vertebra and assessing pain in liver transplant recipients. For LTx patients, it was recommended to work with diverse teams to incorporate dietary and psychological enhancements with HBEP therapies. However, there are variations in the results since every patient is unique and because there are several affecting factors. To fully comprehend the long-term impacts of exercise on post-LT recovery and quality of life, more study is required. Resolving discrepancies and improving comprehension of study findings may be achieved by investigating these variables and possibly modifying research techniques.

## Conclusions

By and large, the finding revealed a substantial impact of therapeutic exercise on patient outcomes in terms of anthropometric characteristics, abdominal pain, HGS, and PMI. The mean PMI at post-therapeutic exercises showed a significant increase by measuring the psoas major muscle at the level of the third lumbar vertebra by abdominal CT, which may indicate the extent of improvement and recovery from the ND sarcopenia in LTx.

The results emphasize the potential advantages of pre- and post-therapeutic exercise in LTx, including enhancements in muscle strength and mass, as well as pain management associated with liver function and general health. Optimizing patient outcomes and promoting a more comprehensive approach to liver disease treatment may be achieved by including exercise activities in pre- and post-LT care procedures.

## References

[1] Sinclair M, Gow PJ, Grossmann M, Angus PW (2016). Review article: sarcopenia in cirrhosis-aetiology, implications and potential therapeutic interventions. Aliment Pharmacol Ther.

[2] Carey EJ, Lai JC, Sonnenday C (2019). A North American expert opinion statement on sarcopenia in liver transplantation. Hepatology.

[3] Wang CW, Feng S, Covinsky KE, Hayssen H, Zhou LQ, Yeh BM, Lai JC (2016). A comparison of muscle function, mass, and quality in liver transplant candidates: results from the functional assessment in liver transplantation study. Transplantation.

[4] Luengpradidgun L, Chamroonkul N, Sripongpun P, Kaewdech A, Tanutit P, Ina N, Piratvisuth T (2022). Utility of handgrip strength (HGS) and bioelectrical impedance analysis (BIA) in the diagnosis of sarcopenia in cirrhotic patients. BMC Gastroenterol.

[5] Nishikawa H, Shiraki M, Hiramatsu A, Moriya K, Hino K, Nishiguchi S (2016). Japan Society of Hepatology guidelines for sarcopenia in liver disease (1st edition): recommendation from the working group for creation of sarcopenia assessment criteria. Hepatol Res.

[6] Aamir T, Iftikhar S, Khan RR, Khan MK (2019). Role of physiotherapy in improving quality of life in liver transplant patients. Rehab J.

[7] Senduran M, Yurdalan U (2012). Physiotherapy in liver transplantation. Liver Transplantation - Technical Issues and Complications.

[8] Ergene T, Karadibak D, Polat KY (2019). Fatigue and physiotherapy in liver transplant recipients. Clin Exp Health Sci.

[9] Roshdy SH, El-Nahas NG, Abd el Hady AA, El Faizy MW (2019). Impact of early pulmonary rehabilitation on post liver transplantation. J Adv Pharm Edu Res.

[10] Dunn MA, Rogal SS, Duarte-Rojo A, Lai JC (2020). Physical function, physical activity, and quality of life after liver transplantation. Liver Transpl.

[11] Dhaliwal M, Raghunathan V (2023). Intensive care issues in post-operative pediatric liver transplantation. Peri-operative Anesthetic Management in Liver Transplantation.

[12] Beekman L, Berzigotti A, Banz V (2018). Physical activity in liver transplantation: a patient's and physicians' experience. Adv Ther.

[13] Yüksel Ergene T, Karadibak D, Dönmez R, Polat KY (2022). Effects of early resistance training after liver transplantation procedures: a randomized controlled pilot trial. Turk J Gastroenterol.

[14] Park SJ, Yoon JH, Joo I, Lee JM (2023). Newly developed sarcopenia after liver transplantation, determined by a fully automated 3D muscle volume estimation on abdominal CT, can predict post-transplant diabetes mellitus and poor survival outcomes. Cancer Imaging.

[15] Aarden JJ, Reijnierse EM, van der Schaaf M (2021). Longitudinal changes in muscle mass, muscle strength, and physical performance in acutely hospitalized older adults. J Am Med Dir Assoc.

[16] Gur A, Kose A, Oguzturk H (2020). Prognostic value of routine biochemistry profile of liver transplant patients admitted to the emergency department with a suspected infection. Front Emerg Med.

[17] Bernardi M (2023). Effective albumin - a novel paradigm in the management of decompensated liver cirrhosis. J Transl Int Med.

[18] Singh V, De A, Mehtani R (2023). Asia-Pacific association for study of liver guidelines on management of ascites in liver disease. Hepatol Int.

[19] Geladari E, Alexopoulos T, Kontogianni MD, Vasilieva L, Mani I, Alexopoulou A (2023). Mechanisms of sarcopenia in liver cirrhosis and the role of myokines. Ann Gastroenterol.

[20] Hammad A, Kaido T, Aliyev V, Mandato C, Uemoto S (2017). Nutritional therapy in liver transplantation. Nutrients.

[21] Williams FR, Vallance A, Faulkner T (2019). Home-based exercise in patients awaiting liver transplantation: a feasibility study. Liver Transpl.

